# The accuracy of auditory spatial judgments in the visually impaired is dependent on sound source distance

**DOI:** 10.1038/s41598-020-64306-8

**Published:** 2020-04-28

**Authors:** Andrew J. Kolarik, Rajiv Raman, Brian C. J. Moore, Silvia Cirstea, Sarika Gopalakrishnan, Shahina Pardhan

**Affiliations:** 1 Vision and Eye Research Institute, School of Medicine, Anglia Ruskin University Cambridge, United Kingdom; 2 Department of Psychology, University of Cambridge, Cambridge, United Kingdom; 3 Shri Bhagwan Mahavir Vitreoretinal Services, Sankara Nethralaya Eye Hospital, Chennai, India; 4 School of Computing and Information Science, Anglia Ruskin University Cambridge, United Kingdom; 5 Faculty of Low Vision Care, Elite School of Optometry, Chennai, India; 6 Low Vision Care Department, Sankara Nethralaya Eye Hospital, Chennai, India

**Keywords:** Perception, Human behaviour

## Abstract

Blindness leads to substantial enhancements in many auditory abilities, and deficits in others. It is unknown how severe visual losses need to be before changes in auditory abilities occur, or whether the relationship between severity of visual loss and changes in auditory abilities is proportional and systematic. Here we show that greater severity of visual loss is associated with increased auditory judgments of distance and room size. On average participants with severe visual losses perceived sounds to be twice as far away, and rooms to be three times larger, than sighted controls. Distance estimates for sighted controls were most accurate for closer sounds and least accurate for farther sounds. As the severity of visual impairment increased, accuracy decreased for closer sounds and increased for farther sounds. However, it is for closer sounds that accurate judgments are needed to guide rapid motor responses to auditory events, e.g. planning a safe path through a busy street to avoid collisions with other people, and falls. Interestingly, greater visual impairment severity was associated with more accurate room size estimates. The results support a new hypothesis that crossmodal calibration of audition by vision depends on the severity of visual loss.

## Introduction

The World Health Organization (WHO) estimated that globally 188.5 million people have mild visual loss, 217 million have moderate to severe losses, and 36 million are blind^1^. It has now been well established that full blindness (total visual loss or light perception only) can result in enhancement of certain auditory spatial abilities and worsening of others (for reviews, see^2–7^). For example, blindness often results in dramatic improvements in echolocation skills^4,8^ and the ability to locate sounds in azimuth (left-front-right judgments)^9,10^, but leads to significantly poorer ability to judge the vertical position of sounds^11,12^, or judge sound position with respect to external acoustic landmarks^13^. It has been suggested that the changes underlying enhanced performance are fundamentally related to adaptations within the occipital cortex, where visual areas of the brain are recruited to process auditory inputs in the event of visual loss^5,14,15^. However, the underlying principles of what drives changes in auditory abilities following visual loss are not well understood. It is not yet known how severe the visual loss needs to be before significant alterations in auditory abilities are observed, or whether the relationship between severity of visual loss and changes in auditory abilities is systematic. If it is systematic, then people with more modest visual losses should exhibit smaller changes in auditory abilities than those with more severe visual losses.

Previous work showed that compared to sighted controls, individuals with total vision loss estimate near sound sources to be farther away and estimate farther sound sources to be closer^16,17^, demonstrating the critical role that vision plays in calibrating auditory space^18^. A number of studies have also shown that partial visual deprivation affects auditory localization abilities. Myopic (short-sighted) participants are more accurate than sighted controls for azimuthal localization^19^ and echolocation, and show greater sensitivity to echoic spatial cues^20^. Myopic and amblyopic participants have been reported to show significantly smaller self-positioning errors using sound to assess their position in a room than sighted controls^21^. Also, partially sighted participants self-reported better azimuthal sound localization and improved abilities to follow speech when switching between different talkers^22^.

Although partial loss of vision entails an increased reliance on hearing for awareness and safety, communication and interaction, and for enjoyment through sound^23^, it is not known how hearing is affected by the severity of partial visual loss. We explored this by obtaining judgments of the distance of sound sources and of room size using sound for participants with a range of severities of visual loss, to test the hypothesis that crossmodal calibration is dependent on the magnitude of the sensory loss. It was hypothesized that systematic increases in auditory judgments of distance and room size would be associated with the severity of visual loss, with greater estimates associated with more severe visual loss. To investigate whether this was true, and whether it generalized across different room environments and stimuli, participants were tested in virtual anechoic and reverberant rooms, using speech, music and noise stimuli. The different stimuli were chosen as they varied in their spectro-temporal characteristics. It was hypothesized that in a virtual reverberant room, estimates of distance and room size would be greater than in the virtual anechoic room, as research suggests that greater reverberation is associated with increased perceived distance^24^ and room size estimates^25,26^ for normally sighted participants. It was hypothesized that estimates would be more veridical for speech^27^ than for noise^28^ or music, as normally sighted participants were previously shown to be able to utilize their familiarity with the acoustic characteristics of speech to give more veridical distance estimates^29^.

## Methods

### Participants

The participants were recruited from Sankara Nethralaya Eye Hospital in Chennai, India. Fifty six participants took part. They were categorized into four groups according to their visual acuities, with category boundaries chosen to include a wide range of visual losses. Group 1 consisted of sighted controls (n = 18, 13 females, mean age 21.1 yrs, range 20–25 yrs, LogMAR [Logarithm of the Minimum Angle of Resolution, used to estimate visual acuity] = 0), group 2 had mild visual impairment (n = 16, 4 females, mean age 21.7 yrs, range 18–31 yrs, LogMAR 0.1–1), group 3 had mid-range visual impairment (n = 12, 7 females, mean age 21.1 yrs, range 17–28 yrs, LogMAR 1.1 to 2.9), and group 4 had severe visual impairment (n = 10, 5 females, mean age 21.9 yrs, range 18–31 yrs, LogMAR 3–4). See Supplementary Table S1 online for a summary of group characteristics. Some of the participants with mild visual impairment wore corrective glasses. For reference, legal blindness in the United Kingdom and United States of America is defined as having a better eye visual acuity when using a corrective lens equal to or worse than LogMAR = 1. One-way ANOVAs showed no significant differences in age between the four groups (*p* > 0.05), no significant differences in duration of visual loss across the groups with visual losses (*p* > 0.05), and no significant differences in age of onset of visual loss across the groups with visual losses (*p* > 0.05). Hearing for all participants was normal or near-normal, as indicated by pure-tone-average better-ear hearing thresholds ≤25 dB HL across 0.5, 1, 2, 4, 6, and 8 kHz^30^. The study and methods followed the tenets of the Declaration of Helsinki, and informed consent was obtained from participants after the nature and possible consequences of participation were explained. Approval for the experiments was given by the Anglia Ruskin University Ethics Panel.

### Apparatus and data acquisition

**Figure 1:**
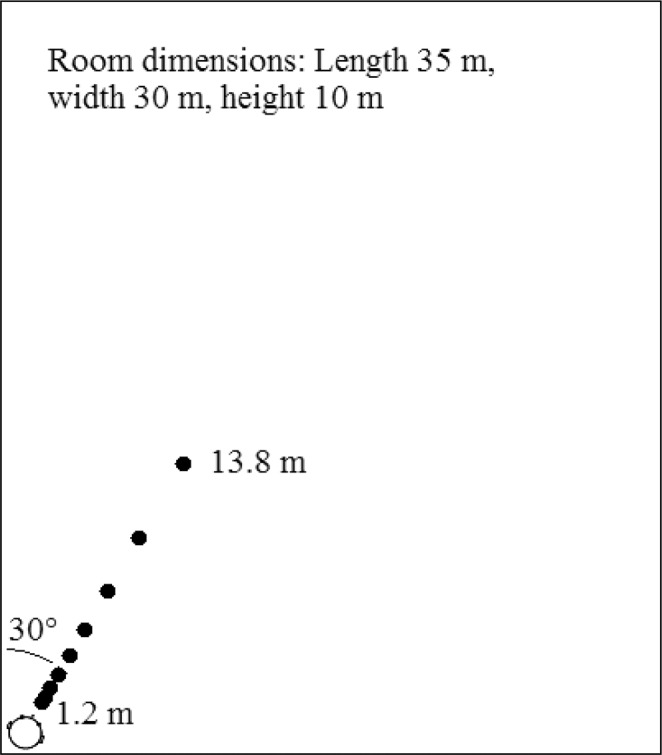
Schematic of the simulated room. The participant was positioned in the lower-left corner at 1 m from each wall, facing forwards at 30° relative to the longer wall. The virtual sound sources (closed circles) were positioned directly forward of the participant between 1.2 and 13.8 m away.

Testing took place in a quiet room. Stimuli were presented using Sennheiser HD219 headphones. The stimuli were generated using methods described in our previous studies^16,17,31–33^. A virtual room measuring 35 m (length) x 30 m (width) x 10 m (height), that was either anechoic (where only sound level information about distance was available^24,28,34^) or reverberant (where both level and echoic direct-to-reverberant energy ratio [DRR] distance cues were available^33,35,36^), was generated using an image-source model (ISM)^37^. The reverberation time T_60_ (the time taken for the sound level to fall by 60 dB) was 700 ms, as used in previous studies^16,35^. The ISM produced a room impulse response (RIR) between a virtual auditory source and a receiver positioned a set virtual distance away. Convolution of the RIR and an auditory stimulus generated a virtual sample of the stimulus heard in the virtual room at the simulated distance. Stimuli were simulated at 1 m height, at 0° elevation and 0° azimuth relative to the front of the head. The participant was in the near-left corner of the room, facing forwards at 30° relative to the longer wall (for a schematic of the simulated room, see Fig. 1).

Participants heard speech, music, and broadband noise stimuli, as used in our previous work^16,31^. Speech stimuli had a duration of 1.5 s, were sampled at 22.05 kHz, and were single sentences spoken at a conversational level by a male, selected randomly from the Bench–Kowal–Bamford corpus^16,31,38,39^. Music stimuli had a duration of 7.3 s, were sampled at 22.05 kHz, and played by a jazz trio of piano, bass and drums^16,31,40^. Broadband (0.6–12 kHz) noise stimuli had a duration of 90 ms and a rise/fall time of 10 ms, and were sampled at 44.1 kHz^10,16,31,32^.

Virtual distances of the sound source were 1.22, 1.72, 2.44, 3.45, 4.88, 6.90, 9.75 and 13.79 m, chosen to match those used in previous work^16,28,31^. Sounds were presented at 65 dB SPL (unweighted) for a simulated distance of 1 m and the level decreased as the virtual distance was increased. Stimuli were spatially rendered by convolving the direct sound component with a non-individualized head-related transfer function (HRTF) using publicly available measurements^41^. These HRTFs were used in previous studies of auditory distance judgments^16,31,32,42^.

### Procedures

Participants wore blindfolds throughout testing^8,43^, and were instructed to imagine that they were seated in a rectangular room of a certain size. They were told that sounds would be played from loudspeakers from a range of distances and they would be required to estimate by verbal report the distance of each sound in meters and centimeters. After hearing a series of sounds, they would be requested to estimate the room size by reporting the length, width, and height of the room in meters and centimeters.

Single sounds were presented in a pseudo-random order at the various simulated distances. At the end of each block of trials (80 stimulus presentations; see below), the participant gave an estimate of the room size. All responses were recorded by the experimenter. Participants did not receive training, were not given feedback, and were not limited in their response time.

For each block of trials, the stimulus type (speech, music, or noise) and room condition (anechoic or reverberant) were held constant. There were 10 repetitions for each of the 8 simulated distances for each block and 3 stimulus types x 2 room conditions = 6 blocks, with 480 trials in total. Data collection occurred in a single session lasting approximately 1 hour and 40 minutes, during which the blocks were presented in a random order.

## Results

### Distance estimates

**Figure 2:**
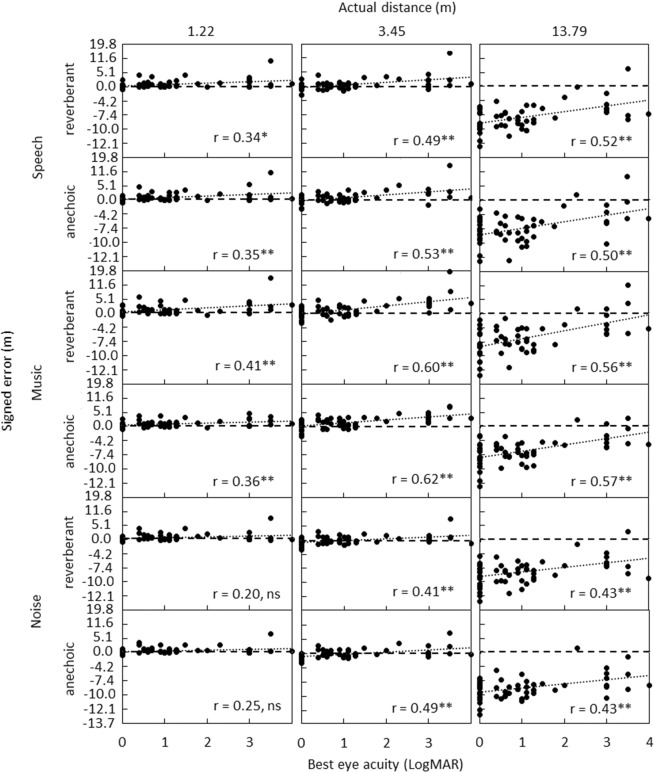
Individual judged distance signed error values plotted against best eye visual acuity. Rows of panels from top to bottom show results for simulated anechoic and reverberant rooms for speech, music, and noise. The left, middle, and right panels show results for a subset of simulated distances of 1.22, 3.45 and 13.79 m, respectively. LogMAR = 0 represents best visual acuity and 4 the worst. Linear fits to the data are shown by dotted lines. Perfect performance is indicated by the long-dashed lines. Pearson r values are reported in the bottom-right corner of each panel (in this and subsequent figures and Table 1, ns = non-significant, * = *p*<0.05, ** = *p*<0.01).

Figure 2 shows scatter plots of signed error values for judged distance against best eye visual acuity, for a subset of the tested distances of 1.22, 3.45, and 13.79 m. Pearson correlation (r) values are reported in the bottom-right corner of each panel and are shown for all distances used in Table 1. Correlations were significant in all cases, with the exception of the distance of 1.22 m for the noise conditions, and 1.72 m for the reverberant noise condition.

**Table 1:** Pearson correlations (r) between judged distance signed error values and best eye visual acuity, for each simulated sound source distance and experimental condition. * = *p* < 0.05, ** = *p* < 0.01.

Simulated distance (m)	1.22	1.72	2.44	3.45	4.88	6.9	9.75	13.79
Reverberant Speech	0.34*	0.40**	0.47**	0.49**	0.51**	0.56**	0.52**	0.52**
Anechoic Speech	0.35**	0.40**	0.47**	0.53**	0.55**	0.55**	0.53**	0.50**
Reverberant Music	0.41**	0.48**	0.58**	0.60**	0.62**	0.62**	0.60**	0.56**
Anechoic Music	0.36**	0.48**	0.59**	0.62**	0.60**	0.61**	0.59**	0.57**
Reverberant Noise	0.20	0.25	0.32*	0.41**	0.46**	0.48**	0.43**	0.43**
Anechoic Noise	0.25	0.28*	0.40**	0.49**	0.40**	0.41**	0.45**	0.43**.

**Figure 3:**
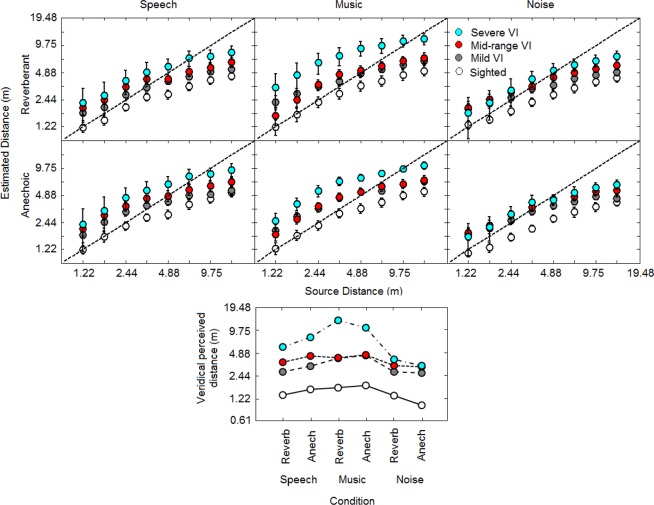
Judged auditory distance plotted as a function of virtual sound source distance (top panels), and veridical perceived distances (distances at which the perceived distance most accurately matched the simulated distance) for each condition (bottom panel). In the top panels, geometric mean estimates are indicated by open (sighted participants), and closed grey (mild VI), red (mid-range VI), and blue (severe VI) symbols. The left, middle and right panels show results for speech, music and noise stimuli, respectively. The upper and lower panels show results for the reverberant and anechoic rooms, respectively. Perfect performance is indicated by the dashed lines. Error bars represent ±1 standard error across participants, and are not shown when smaller than the symbol size. The lower panel shows veridical perceived distances in each condition for each group; values were obtained from the intercept between lines of best fit for each condition (not shown) and the line for perfect performance in the top panels.

The top panels of Fig. 3 show judged auditory distance plotted as a function of source distance for each condition, on log-log co-ordinates. Geometric mean estimates (following previous work^28^) are indicated by open symbols for sighted participants and filled symbols for VI participants. Consistent with the literature^7^, the sighted group judged sound source distance most accurately for the closest sounds and least accurately for farther sounds. On average, the severe VI group showed the opposite pattern, judging sound source distance most accurately for farther sounds and least accurately for closer sounds. All VI groups estimated the sound source distances to be farther than did the sighted controls. Estimated distance systematically increased with severity of visual loss, with the severe VI group on average judging sounds to be twice as far away as for the sighted controls. For statistical comparison purposes, and similar to the procedure used by Voss *et al*.^44^ for analyzing spatial estimates, the distances tested were grouped into near (1.22–1.72 m), middle (2.44–4.88 m), and far (6.90–13.79 m) distances. Near space was previously described by Zahorik, *et al*.^45^ as less than 1.9 m from the participant, and it includes the region where tactile feedback might be used to calibrate audition as an alternative to visual feedback. Grouping the distances also allowed us to compare the results to previous work showing that absolute judgments of distance for speech and music were relatively accurate at near and middle distances and less accurate at far distances^16,29^. A mixed-model ANOVA on the logarithms of the estimated distances with distance, reverberation time (i.e. anechoic vs. reverberant virtual room) and stimulus as within-subjects factors and visual status as a between-subjects factor showed main effects of visual status (*F*(3, 52) = 6.6, *p* < 0.001), distance (*F*(2, 104) = 303.5, *p* < 0.001), and stimulus (*F*(2, 104) = 60.0, *p* < 0.001), and significant interactions between stimulus and visual status (*F*(6, 104) = 7.61, *p* < 0.001), distance and visual status (*F*(6, 104) = 4.81, *p* < 0.001), stimulus and distance (*F*(4, 208) = 34.9, *p* < 0.001), stimulus and reverberation time (*F*(2, 104) = 9.9, *p* < 0.001), stimulus reverberation time and visual status (*F*(6, 104) = 2.7, *p* < 0.05), and stimulus and reverberation time and distance (*F*(4, 208) = 2.9, *p* < 0.05). No other main effects or interactions were significant (all *p* > 0.05). Post hoc tests with Bonferroni correction showed that in all conditions the severe VI group judged sounds to be significantly farther than the sighted group, except for the speech and noise stimuli at near distances. The mid-range VI group judged sounds to be significantly farther than the sighted group in all conditions for near and middle distances only, except for the reverberant music stimuli, and the anechoic music stimuli at near distance only. The mild VI group judged sounds to be significantly farther than the sighted group for anechoic music and noise stimuli at near and middle distances only.

The bottom panel of Fig. 3 shows veridical perceived distances, which are the distances at which the perceived distance matched the simulated distance, for each stimulus condition and severity. Values were obtained from the intercept between lines of best fit for each condition (not shown) and the lines showing perfect performance in the top panels. Smaller values of veridical distance indicate that the distances at which the reported distance and simulated distance matched were nearer to the participant. Veridical perceived distances increased as severity of visual loss increased, with the farthest veridical perceived distances occurring for the group with severe visual impairment in anechoic and reverberant music conditions. These results show that groups with severe visual losses judged farther sound distances most accurately than sighted participants, whereas the opposite was the case for close sounds.

### Room size estimates

**Figure 4:**
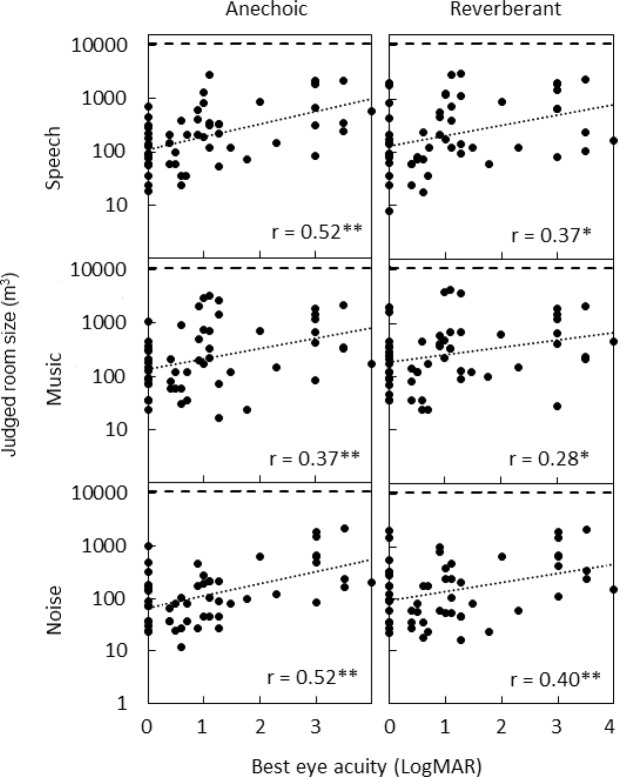
Individual judged room size estimates plotted against best eye visual acuity. Rows of panels from top to bottom show results for speech, music, and noise. The left and right columns show results for anechoic and reverberant simulated rooms, respectively. Linear fits to the data are shown by dotted lines. Veridical room size is indicated by the long-dashed lines (room size = 10500 m^3^). r values are shown in the bottom-right corner of each panel.

Figure 4 shows individual room size estimates plotted against best eye visual acuity. r values are shown in the bottom-right corner of each panel. The room size was consistently underestimated. Correlations between room size estimates and best eye visual acuity were positive and significant for all conditions (*p* < 0.05), indicating that participants with greater visual losses made larger room size estimates.

## Discussion

The results show that the greater the severity of visual loss, the larger the auditory judgments of distance and room size, with the largest perceived distances and room sizes associated with the most severe visual impairment. On average the group with severe visual losses judged sounds to be twice as far away, and rooms to be three times larger, than sighted controls. For normally sighted participants, auditory space is assumed to be calibrated primarily by vision^46^, which is considerably more accurate than audition^47–49^. Internal representations of auditory space are constantly updated using motor and visual feedback to align auditory and visual representations of space^50^. This leads to the prediction that altering the accuracy of the visual signal will affect how auditory space is calibrated. The results demonstrate that full blindness is not necessary for judged auditory distance and room size to be affected by visual loss, and that changes in auditory perception are systematic and related to the severity of visual loss. The current results support the hypothesis that crossmodal calibration is dependent on the magnitude of the sensory loss. Consistent with this idea, the visually impaired participants, who lacked an intact visual signal to calibrate auditory space accurately, perceived sound sources as being at greater distances than sighted controls and judged the room to be larger. As a consequence, visually impaired individuals show a deficit in accuracy for judging the distance of closer sounds. However, it is in this region of space that accurate judgments of distance are required to guide rapid motor responses in order to react to auditory events, particularly threatening or interesting sounds^51^. For example, the increase in perceived distance of a sound source would make it harder for somebody with visual loss to plan a safe path through a busy street to avoid collisions and falls.

On average, normally sighted controls made more veridical judgments for stimuli that were near 1 m, and systematically underestimated the distance of sound sources that were farther away, agreeing with previous research conducted in real and virtual rooms^7,45^. Consistent with previous work^16^, estimates by normally sighted controls tended to be more veridical for speech and music than for noise stimuli, for which judgments tended to be most accurate for sounds at near and middle distances. For speech stimuli, normally sighted controls were most accurate for near and middle distances, and less accurate for far distances, as observed in a previous study^29^. However, it is possible that the relatively long durations of the speech and music stimuli, which were chosen to match those used in previous work, may have affected the results. This needs further examination. Similar performance was found for anechoic and reverberant conditions, suggesting that the presence of the DRR cue did not affect spatial judgments for all groups of participants. This may be due to the comparatively short room reverberation time used in the study (700 ms), as previous studies have shown that increasing the room reverberation time increases perceived distance judgments^24^ and room size estimates^25,26^ for normally sighted participants. This also needs further examination.

The current study investigated how judged auditory distance and room size were affected by visual loss when both auditory level and DRR cues were present (reverberant condition) and when the level cue in isolation was available (anechoic condition). Although level is generally the dominant cue used for making distance judgments, DRR has been shown to be as effective as level when making distance judgments in highly reverberant environments for farther source distances for normally sighted participants only^33^. Also, fully blind participants often develop enhanced abilities to process echoes^32,52^. Further work is needed to establish whether severity of visual loss affects the ability to process the DRR cue in isolation. By making DRR the only useful distance cue, for example by equalizing or roving the overall stimulus level, it would be possible to examine whether the severity of visual loss is correlated with the ability to process reverberation information when making distance judgments.

Another area for future work regards the relevance of the onset of visual deprivation and the duration of visual loss for crossmodal plasticity and calibration. Full visual loss in early life during a critical period of development is likely to affect the spatial abilities of the remaining senses^53^. This is the basis of the crossmodal calibration hypothesis, that loss of vision at an early age affects the spatial calibration of the remaining senses^47^. Investigations into the influence of the age of onset of visual loss on audition have generally been confined to testing participants with full blindness^54^. Further work is needed to establish how the onset of visual loss affects the spatial processing abilities of individuals with partial visual losses.

Absolute judgments of auditory distance require well-calibrated mapping between internal spatial representations and the distance to external sound-producing objects^7^. Auditory representations of distance are likely to be relatively coarse compared to visual representations^16,55,56^, and the calibration of internal auditory spatial maps is generally thought to be realized using visual signals, due to the greater accuracy of visual spatial information than auditory information^46^. Tactile feedback in peripersonal space might be used to calibrate audition as an alternative to vision^11^, and audiomotor feedback from the systematic changes in sound cues arising from the movement of the head and body has also been suggested as a means to calibrate internal representations of sound azimuth in the absence of vision^12^. In contrast to the relatively accurate localization performance for azimuth observed in blind individuals^9,42^, the current results for partially sighted participants are consistent with previous work reporting that absolute distance accuracy is worse for blind than for sighted controls in peripersonal space^16,17,57,58^. Taken together, these results suggest that if vision is absent or degraded, tactile feedback is insufficient to calibrate auditory distance in peripersonal space, corresponding to “near” space in the current results. The ability to judge the distance of a single, static sound source is relatively poor compared to the ability to judge the azimuth of a sound in normally sighted individuals^59^ as well as blind individuals^5,16^. Although it is unclear why tactile and audiomotor feedback appear to be sufficient to calibrate internal representations of sound azimuth but not distance, one possible explanation is that small changes in sound azimuth cues are easier to detect than small changes in auditory distance cues, rendering azimuth cues but not distance cues amenable to calibration by tactile or audiomotor feedback. However, further investigation is needed. The current results suggest that partial visual loss affects the calibration of internal representations of auditory distance, and that the magnitude of change in the internal spatial representations of distance stemming from visual loss is dependent on the severity of visual loss.

A potential limitation of the experiment concerns the choice of response measure (verbally stating an estimated distance). The use of this measure depends on the participants’ ability to judge distance in standard measurement units. This raises the possibility that differences between groups may not be related to auditory perception, but rather to the ability to accurately estimate distance. It is possible that the outcomes would have differed if a more objective and potentially less biased spatial measure was used^60,61^. However, we have previously shown that visual loss does not affect distance judgments using a non-auditory and non-visual task, in which blind participants and sighted controls were required to walk over various distances (2, 5, or 10 m). Performance was similar across groups, indicating that the general ability to estimate distances was similar for sighted and fully blind participants^16^. These results suggest that visual loss does not affect the ability to estimate distance. Measurements such as walking to the perceived location of the sound source have been used as an alternative to reporting perceived distance^49^. Loomis *et al*.^49^ reported that judgments of auditory distance by normally sighted participants made using verbal report or walking responses were generally concordant, with less variability associated with the walking responses, suggesting that the findings of the current study would likely be similar if walking responses instead of verbal responses were used to measure perceived auditory distance.

The literature indicates reasonable agreement between subjective and objective measures of auditory perception for partially sighted participants^22^, suggesting that changes in audition shown under laboratory conditions can reflect judgments in real-life situations. Thus, the effects of degree of visual loss on distance and room size judgments shown here would probably be associated with differences in the ability to use sound effectively when navigating through the world. A key open question regarding the effect of sensory deprivation on the remaining senses is how residual vision may affect the development of cross-modal plasticity^54^. For individuals with remaining pattern vision, like those who took part in the current study, competitive visual inputs may affect the degree to which cross-modal recruitment occurs. The current results suggest that changes in auditory distance and room size perception are systematically related to the severity of visual impairment, raising the possibility that cross-modal recruitment may also be proportional to the severity of visual impairment. This remains to be investigated.

## Supplementary information

Supplementary Table S1.

## Data Availability

The datasets generated during and analyzed during the current study are available from the corresponding author on reasonable request.
