# Supplementary material for: The accuracy of auditory spatial judgments in the visually impaired is dependent on sound source distance
Source: Sci Rep. 2020 Apr 28;10:7169. doi: 10.1038/s41598-020-64306-8 (PMC7189236; doi:10.1038/s41598-020-64306-8)
Supplement: Supplementary file 1 — Supplementary Table S1. [file 41598_2020_64306_MOESM1_ESM.docx]

The accuracy of auditory spatial judgments in the visually impaired is dependent on sound source distance

Andrew J. Kolarik, Rajiv Raman, Brian C. J. Moore, Silvia Cirstea, Sarika Gopalakrishnan, Shahina Pardhan

| Cause of vision loss, participant group | Age (yrs) | Age of onset of visual loss (yrs) | Duration of visual loss ( yrs) | Better-eye visual acuity (LogMAR) |
| --- | --- | --- | --- | --- |
| Hypoplastic Disc, 2 | 25 | Birth | 25 | 0.40 |
| Retinitis Pigmentosa, 2 | 20 | 12 | 8 | 0.40 |
| Neuritis, 2 | 23 | 1 | 22 | 0.40 |
| Stargardt's, 2 | 24 | 6 | 18 | 0.50 |
| Bietti's crystalline dystrophy, 2 | 31 | 4 months | 31 | 0.50 |
| Retinitis Pigmentosa, 2 | 21 | Birth | 21 | 0.60 |
| Retinitis Pigmentosa, 2 | 19 | 17 | 2 | 0.60 |
| Retinitis Pigmentosa, 2 | 20 | Birth | 20 | 0.60 |
| Marfan Syndrome, 2 | 20 | 10 | 10 | 0.70 |
| Cone rod dystrophy, 2 | 18 | Birth | 18 | 0.70 |
| Nystagmus, 2 | 20 | Birth | 20 | 0.90 |
| Amblopia, 2 | 18 | Birth | 18 | 0.90 |
| Retinitis Pigmentosa, 2 | 25 | 14 | 11 | 0.90 |
| Stargardt's, 2 | 21 | Birth | 21 | 1.00 |
| Cone dystrophy, 2 | 18 | 10 | 8 | 1.00 |
| Hypoplastic Disc, 2 | 24 | Birth | 24 | 1.00 |
| Retinitis Pigmentosa, 3 | 18 | Birth | 18 | 1.10 |
| Stargardt's, 3 | 23 | Birth | 23 | 1.10 |
| Retinitis Pigmentosa, 3 | 18 | 11 | 7 | 1.10 |
| Healed retinitis, 3 | 23 | Birth | 23 | 1.10 |
| Stargardt's, 3 | 20 | 17 | 3 | 1.28 |
| Retinitis Pigmentosa, 3 | 20 | 4 | 16 | 1.28 |
| Cone rod dystrophy, 3 | 18 | Birth | 18 | 1.28 |
| Leber congenital amaurosis , 3 | 22 | Birth | 22 | 1.28 |
| Stargardt's, 3 | 27 | Birth | 27 | 1.48 |
| Retinitis Pigmentosa, 3 | 28 | 6 | 22 | 1.77 |
| Pale disc, 3 | 17 | Birth | 17 | 2.00 |
| Pale disc, 3 | 19 | 1.5 | 17.5 | 2.30 |
| Retinitis Pigmentosa, 4 | 19 | Birth | 19 | 3.00 |
| Optic atrophy, 4 | 26 | 3 | 23 | 3.00 |
| Optic atrophy, 4 | 18 | Birth | 18 | 3.00 |
| Glaucoma, 4 | 31 | Birth | 31 | 3.00 |
| Retinal detachment, 4 | 20 | 4 | 16 | 3.00 |
| Glaucoma, Aphakia, Pseudophakia, 4 | 18 | Birth | 18 | 3.00 |
| Retinitis Pigmentosa, 4 | 22 | 5 | 17 | 3.50 |
| Phthisical eye, 4 | 19 | Birth | 19 | 3.50 |
| Corneal opacity, 4 | 22 | Birth | 22 | 3.50 |
| Total corneal opacity, 4 | 24 | Birth | 24 | 4.00 |

Supplementary Table S1. Summary of characteristics of participants with visual loss, including causes of visual loss and participant group (Group 2: mild visual impairment; Group 3: mid-range visual impairment; Group 4: severe visual impairment), age, age of onset of visual loss, duration of visual loss, and better-eye visual acuity.
